# Supplementary material for: Flare differentially rotates sunspot on Sun's surface
Source: Nat Commun. 2016 Oct 10;7:13104. doi: 10.1038/ncomms13104 (PMC5062561; doi:10.1038/ncomms13104)
Supplement: Supplementary Information — Supplementary Figure 1. [file ncomms13104-s1.pdf]

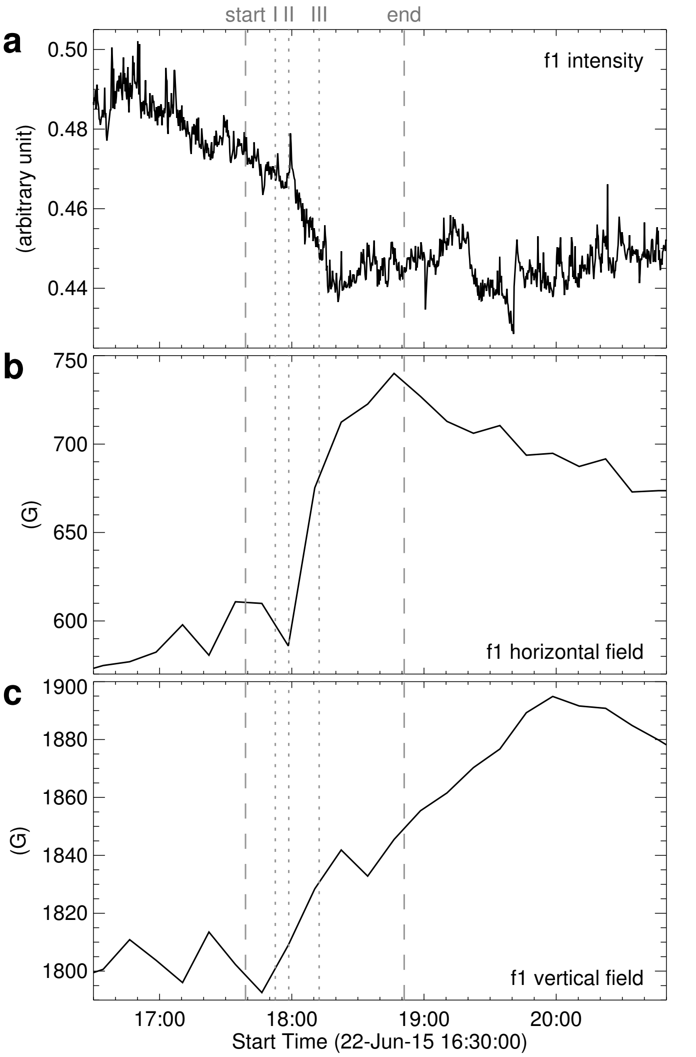

Supplementary Figure 1 | Evolution of $f1$ region properties. (**a**) Mean normalized TiO intensity. (**b**) Mean horizontal field. (**c**) Mean vertical field. The noise level of SDO/HMI horizontal and vertical field is roughly 100 and 10 G, respectively. The vertical dashed lines mark the start and end times of the flare in GOES 1.6–12.4 keV SXR flux, and the dotted lines mark the three main Fermi 25–50 keV HXR peaks I–III.
